# Exploring Clinical Practice and Developing Clinician Self-Reflection Through Cross Self-Confrontation Methodology: An Application Within an Addiction Medicine Unit

**DOI:** 10.1177/23333936211054800

**Published:** 2021-11-03

**Authors:** Sophie Paroz, Jean-Bernard Daeppen, Martine Monnat, Michael Saraga, Francesco Panese

**Affiliations:** 1 30635 Lausanne University Hospital and University of Lausanne, Lausanne, Switzerland; 2 Public Health Service of Canton of Vaud, Lausanne, Switzerland

**Keywords:** qualitative methodology, cross self-confrontation, addiction, substance use disorders, addiction-related clinical practice, reflective practice, Switzerland

## Abstract

Use of the methodology of cross self-confrontation (CSC) is limited in the field of healthcare and in the context of clinical practice. We applied this methodology within an addiction medicine unit of a university hospital, as part of an exploration of addiction-related clinical difficulties. Cross self-confrontation was used according to a 3-phase design based on video recorded clinical interviews with pairs of nurses and medical doctors. The article reports and discusses the application of CSC in a specific clinical context and illustrates the methodological process through one result. Findings suggest two major strengths of CSC in the context of clinical practice research and education: (1) the capacity to elicit tacit knowledge from daily clinical practice and (2) the ability to enhance self-reflection by questioning professionals both individually and collectively. Further use of CSC in nursing surroundings and clinical settings should be encouraged.

## The Call of Cross Self-Confrontation Methodology to Explore Clinical Practice

The social and human sciences are a constant source of inspiration for developing new approaches that best meet the fields of investigation of healthcare research. We turned our attention on a method that emerged in work psychology within the Activity Clinic approach (Clot, 1999, 2009; Clot & Kostulski, 2011), the methodology of cross self-confrontation (CSC) (Clot, 1999; Clot et al., 2000; Kloetzer, 2018). The intention of the various methods proposed by the Activity Clinic is to support the professionals through the analysis and development of their professional activity. Rooted in Vygotsky’s works, the theoretical framework assumes that access to lived experience requires *indirect methods* based on the repetition of the experience (Clot, 2011; Vygotski, 1926/2010). To achieve this, the general methodological strategy rests on shifting the professional activities to a new context and using dialogical tools (Clot, 2011). The methodology of CSC uses this strategy.

Basically, CSC consists of inviting professionals to comment on sequences of their own video recorded activity. In simple self-confrontation interviews (simple SCI), the professional comments on the activity in the presence of a researcher. In cross self-confrontation interviews (cross SCI), the professional performs the same task in the presence of a researcher and another professional. In the original theoretical approach, simple SCI and cross SCI are the second part of a 3-phase process that starts with the constitution of an analysis group and ends with extended analyses by the original analysis group. Cross self-confrontation refers to the full process, although the method is mainly known for its second phase and the successive application of simple SCI and cross SCI.

Cross self-confrontation has been used in many professional contexts, ranging from technical diving (Kloetzer, 2013) to preaching in homily (Kostulski & Kloetzer, 2014) and car manufacturing (Kloetzer, 2018; Quillerou-Grivot & Clot, 2013). But its application within the fields of nursing and medicine is currently limited. One study examined the work conditions of nurses and assistant nurses in a geriatric care unit (Litim, 2006). Another explored changes in the profession among medical radiology technicians. (Pires Jorge, 2016). Finally, an interesting study was conducted in the context of robotic surgery and evolving technology (Seppänen et al., 2017) in an attempt to develop learning strategies for surgeons of a hospital oncology unit. These studies focused on healthcare professions or practices in evolving professional contexts. Unlike previous research using CSC within the field of healthcare, we applied CSC to explore clinical practice in the context of intersubjective activity between clinician and patient.

Our use of CSC was part of a qualitatively-driven multiple-method study (Morse, 2003) that explored clinical addiction practice and related difficulties with different groups of hospital-based clinicians involved in care for patients with substance use disorders (to be published). Firstly, our interest in using CSC was to explore clinical practice through audiovisual data of professional activity and to trigger the thinking from the activity itself. By relying on video footage and requiring professionals to put their action into words, CSC was a way to “narrow the focus to what people are doing” and collect field-grounded knowledge about addiction clinical practice and related challenges (Barry, 2002, p. 1094). Secondly, we intended to take advantage of the reflective process at the heart of CSC to produce scientific knowledge. Exploring clinical practice uncertainty (Fox, 1957; Mackintosh & Armstrong, 2020) and addiction-related clinical practice complexity (O'Connor et al., 2014) requires methods capable of questioning the knowledge, values, and representations that underpin daily practices. Cross self-confrontation appeared to be one of these methods. Finally, the performativity induced by CSC reflective process was of specific interest from a learning perspective and reminiscent of specific clinical educational contexts such as clinical supervision (Falender & Shafranske, 2014; Owen & Shohet, 2013). Cross self-confrontation’s ability to reflect on one’s actions in order to engage in a process of continuous learning and develop professional competencies was demonstrated in various environments (Kloetzer et al., 2018; Seppänen et al., 2017). Being able to observe the educational dimension in a setting with a long tradition of collaborative learning was an appealing concept. By placing professionals in a situation of activity through audiovisual data and encouraging reflective capacity, we expected CSC to allow for a more in-depth analysis of clinical practice than typical qualitative data methods in medical research, such as semi-structured interviews and participant observation, and usual clinical educational contexts.

This article reports the application of this method and discusses its relevance in exploring and developing clinical practice. We present the methodology of CSC as applied within an addiction medicine unit. The adapted design will be shown, specific methodological steps will be detailed, the method will be illustrated through one specific result, and the conclusion will show several challenges encountered and comments addressing the application of CSC to research and education in clinical settings.

## The Application of CSC Within an Addiction Medicine Unit: General Design and Material

### Recruitment and Sample

According to CSC, participants were included by pairs of two and professionals of each pair were of the same profession and same hierarchical level. Recruitment took place in the Addiction medicine unit of a Swiss university hospital. Eligibility criteria included the following: being a nurse or a medical doctor; having a clinical practice involving clinical interviews with patients; and declaring interest in analyzing own practice. In addition, professionals had to meet the requirements of agreeing to video record a clinical interview, being available for a time-consuming project, and feeling comfortable pairing with a given colleague. Team leaders were mobilized to submit a list of potential participants. The leading researcher assessed motivation and requirements fulfillment during individual interviews.

Three pairs of clinicians took part in the research. Pair 1 included two senior nurses of the liaison team, Pair 2 comprised two psychiatry residents of the outpatient clinic, and Pair 3 reunited two junior nurses of the opioid maintenance therapy program. Pairs were limited to three due to the volume of collected data.

### Design

The design implemented for this research (Table 1) was inspired by the Activity Clinic 3-phase design. A research group was constituted in a preliminary phase (Table 1, Pre-phase). It included medical doctors (two senior internist physicians and a senior psychiatrist) and researchers (a qualitative researcher in addiction medicine and a researcher in social studies of medicine) who shared an interest in exploring addiction-related clinical practice. General methodological options, including technical and ethical considerations related to the implementation of CSC in a clinical environment, were discussed in the preliminary phase. Then a 3-phase process was repeated for each recruited pair of clinicians.

**Table 1.** The adapted 3-phase design.

Pre-phase			
	Constitution of a research group		
	Development of general design		
	Definition of fields of investigation		
Phase 1	DEVELOPING		
1A	Research group meeting		Constitution of the pair of professionals Definition of target activity and implementation modalities
1B	1st individual interview with Clinician 1	1st individual interview with Clinician 2	
1C	2nd individual interview with Clinician 1	2nd individual interview with Clinician 2	
Phase 2	VIDEOING AND CONFRONTING		
2A	Clinical interview 1	Clinical interview 2	Video recording of target activity
2B	Video montage of Clinical interview 1	Video montage of Clinical interview 2	Video editing of clinical interviews
2C	Simple SCI with Clinician 1	Simple SCI with Clinician 2	Video recording of confrontation of each professional to own activity
2D	Cross SCI with Clinician 1 and Clinician 2		Video recording of confrontation of each professional to own activity in the presence of a colleague
2E	Video montage of preliminary results		Video editing of complete video recorded material
Phase 3	RESTITUING		
3A	Group interview with Clinician 1 and Clinician 2		Restitution of preliminary results to the pair Further analysis and results approval
3B	Final video montage of results		Video editing of results based on group discussion
3C	Feedback questionnaire		Participants’ individual feedback
3D	Research group meeting		Results restitution to the research group Results discussion
Post-phase	Complementary research group meetings: Pooling of results and further analysis		

Simple SCI = simple self-confrontation interviews; Cross SCI = cross self-confrontation interviews.

Phase 1 included a research group meeting (Table 1, step 1A) and two individual interviews between the leading researcher and each clinician of one pair, that is, Clinician 1 (C1) and Clinician 2 (C2) (Table 1, steps 1B and 1C). The purpose was to identify the activity to work on, anticipate organizational and technical implementation modalities, and constitute the pairs.

Phase 2 was devoted to videoing and confrontation interviews. Each clinician video recorded a full clinical interview (Table 1, step 2A). They were free to record any follow-up interview with any consenting patient presenting a substance use disorder that met the following criteria: no medical contraindication (e.g., severe pain or acute psychiatric disorders); no contraindication related to substance abuse (e.g., under the influence of drugs/alcohol or acute withdrawal symptoms); and sufficient command of the French language. Once the material was recorded, the researcher made a video montage out of selected sequences (Table 1, step 2B; see p. 9–10 for more information) for both clinical interviews. Video montages were used separately for each simple SCI (Table 1, step 2C; see p. 10 for more information) and then jointly for cross SCI (Table 1, step 2D; see p. 11 for more information). At this stage, to conclude Phase 2 and highlight preliminary results, the researcher edited a new video montage out of sequences from all recorded video material (Table 1, step 2E; see p. 12 for more information).

Phase 3 included results discussion, approval, and restitution. The third video montage was discussed as part of an audio recorded group interview gathering both clinicians and the leading researcher (Table 1, step 3A), and was finalized accordingly by the researcher (Table 1, step 3B). To complete the results, a brief qualitative questionnaire was sent to C1 and C2 following the group interview (Table 1, step 3C; see p. 12 for more information). Finally, extracts of the final video montage were reported by the leading researcher to the research group and further discussed (Table 1, step 3D). The full set of results of Pair 1, Pair 2, and Pair 3 were examined among the group of researchers in a complementary phase (Table 1, Post-phase). The 3-phase process for each pair lasted 4–5 months. The full process occurred between September 2017 and July 2019.

### Collected Data

Empirical data are of various types. They include written notes from six preliminary individual interviews; six video recorded clinical interviews and related verbatim transcriptions; six video montages of clinical interviews; six video recorded simple SCIs and related verbatim transcriptions; three video recorded cross SCIs and related verbatim transcriptions; three audio recorded group interviews and related verbatim transcriptions; qualitative data extracted from six questionnaires; and six video montages of pre-final and final results. Notes from six research group meetings and a complementary logbook complete the materials.

### Ethics

On account of the use of material from clinical interviews, a study summary was submitted to the Cantonal Research Ethics Committee for approval (Req-2017-00238). On April 11, 2017, it was decided that the research did not fall within the scope of application of the law on Research on Human Subjects and did not need to be submitted as part of the complete procedure. But at the request of the committee, a series of measures were taken to protect clinicians and patients. All information and consent sheets were submitted and validated by the committee.

## The Application of CSC Within an Addiction Medicine Unit: A Few Steps in Details

This section provides additional information on specific methodological steps and our application of CSC. The numbering of the steps refers to Table 1.

### Steps 2A and 2B: Recording and Editing Clinical Interviews

The recorded video activity is a full clinical interview, which features a clinician and a patient in dialogue. The clinical interviews were video recorded in the absence of researcher or camera operator in order to limit interference in clinical work. Video equipment was made available to clinicians so they were free to record at any time; there was no time limit. The recording was taken to the researcher, along with a brief questionnaire detailing the date and context of the clinical interview, the rationale for choosing that particular interview, and the description of any moment of the interview the clinician wanted to work on.

Original clinical interviews lasted 24–51 min. The researcher fully viewed each clinical interview twice. Then, she made an interview summary and listed covered topics (e.g., risk taking, pharmacology, life history, and withdrawal symptoms) and meaningful activities of the clinician (e.g., note taking, laughing or interrupting the patient). After that, the researcher shortened recordings of clinical interviews for use in simple SCIs and cross SCIs. The main intention was to transform the recorded work activity into an appropriate time format for 90-min encounters. Two clinicians asked for given sequences they specifically wanted to comment. Other sequences were selected by the researcher. Based on the general assumption that any moment of a clinical interview is worth analysis, selection criteria were quite flexible but each video montage had to be representative of the various moments and topics covered in each clinical interview. The reasons for those choices were documented for use in later analyses. The video montages contained 4–6 sequences and lasted 10–12.5 min. The full clinical interview was made available during simple SCI and cross SCI in case clinicians wanted to review and discuss sequences complementary to the selection.

### Step 2C: Simple SCI

The general intention of simple SCIs is to engage professionals in commenting on their own video recorded activity and in explicitly describing the reasons and conditions of what they are doing. The procedure was faithful to the original method. First, the clinician watched the video montage of own clinical interview without interruption. Then, the clinician watched it a second time with these instructions: “Look and comment on what you do and how you do it. Show me what is important to you, challenges you, or surprises you when you see yourself working. You can stop the viewing as soon as something catches your attention.” During the second viewing, the researcher stated reasons for selecting each sequence. The researcher constantly centered the dialogue on the recorded activity. Interventions were limited to rephrasing the instructions, or possibly asking for clarification of a statement. To restore attention to the activity, the researcher would restart the video. Before and after the second viewing, the researcher checked whether the clinician wanted to review other sequences of the initial clinical interview. Two clinicians asked to watch additional material to illustrate specific items of the discussion.

Simple self-confrontation interviewss were video recorded and lasted 56–81 min. The clinician was seated next to the display that was playing the video montage and was filmed from the front.

### Step 2D: Cross SCI

The general intention of cross SCI is to establish a dialogue between professionals and increase their awareness of alternative ways of practicing, so as to facilitate the emergence of professional controversies (Clot, 2005; Kostulski & Kloetzer, 2014). Still in line with the original method, cross SCI began with uninterrupted successive viewing of both video montages of clinical interviews. During the second viewing, the researcher invited each professional to comment on the colleague’s activity as follows: “You will see your colleague’s video recorded clinical interview. You can stop the viewing as soon as something questions you, surprises you or is not clear to you.” The researcher limited the interventions to questions aimed at enhancing the dialogue (e.g., What leads you to ask this question? What do you think of what your colleague says?).

Cross self-confrontation interviewss were video recorded and lasted 86–103 min. The two clinicians were seated on both sides of the display that was playing the video montages and were filmed from the front.

### Steps 2C, 2D, 2E and 3A: Data Analysis

Data collection and analysis are fully articulated in CSC. By having professionals interpret their own clinical activity or that of their colleague, or by intervening on researcher’s preliminary results, clinicians fully contributed to the analysis process throughout the different interview settings. The researcher was responsible for outlining preliminary results from recorded simple SCIs and cross SCIs based on an analysis protocol: (a) first viewing and writing an interview summary, (b) second viewing and describing the content of every intervention (i.e., moments during simple SCI or cross SCI when the clinicians stop the viewing to comment), (c) third viewing and open coding (Strauss & Corbin, 1990), and (d) complementary reading of the interview transcripts. Then we highlighted the following: recurrent topics and expressed difficulties; discrepancies between the selection criteria of the sequences and the topics covered when discussing the sequence; the evolution of the discourse; and apparent contradictions.

This stage of the analysis resulted in extracting emerging themes. A theme was defined as *a specific difficulty encountered in addiction-related clinical practice, associated clinical challenges and any potential clinical responses to face it*. A video montage of sequences from clinical interviews, simple SCIs and/or cross SCIs illustrated each theme. A written description of the different sequences that constituted a theme completed the montage. At this stage of the analysis, the researcher discussed each emergent theme in an audio recorded group interview. Guiding questions were as follows: Should this theme be part of the results? How could you clarify the related clinical challenges? Should we label the theme differently? Is there any other theme that should be part of the results? This stage of analysis resulted in a final editing that integrated clinicians’ input.

### Step 3C: Feedback Questionnaire

A 5-item questionnaire focusing on the participants’ experience and the impact of the experience on practice was added to the design. Questions were as follows: What is your general feedback on this group research experience? What particular moment would you relate? To what extent have you thought about simple SCI/cross SCI in the context of your practice? Can you tell me about a clinical interview that has happened differently since this experience? To what extent do you relate simple SCI/cross SCI and clinical supervision sessions? The questionnaire was self-administered within 1 week of the group interview.

## An Illustration, Step by Step

In the present section, we illustrate each methodological step with qualitative material. To achieve this, the section focuses on one unique theme that emerged during the process: *disgust*. This illustration is based on the experience of Pair 2. It was chosen since it exemplifies well the emergence of a theme through the steps and through a constant attention to the recorded work activity. Extracts from a clinical interview and the various types of interviews that followed were used to illustrate the process.

In our research, themes emerged in various dialogical configurations. In the illustration presented below, dialogue starts in cross SCI with a compliment on a specific activity that departs from the initial reasons for selecting the sequence and the object of discussion during simple SCI. The compliment, by promoting one way of doing things, initiates the discussion. Then, by going back and forth to the initial activity and the trigger compliment, we assist in the “construction of a common object which was not given a priori by the film of the activity” (Kloetzer & Henry, 2010, p. 59), disgust. Little by little, we move from the recognition of *disgust in a specific clinical situation* to the recognition of *disgust as a common addiction-related clinical difficulty*. Progressively, we move from the initial mention of physical disgust to its moral dimension and related clinical challenges. As the process goes on, awareness of disgust emerges, paving the way for alternative ways of thinking or acting in future daily activities.

### Step 2A: Clinical Interview

Our illustration is based on the first sequence of a follow-up clinical interview with a 56-year-old man with an alcohol use disorder. The patient (P) visits the addiction outpatient clinic for the third time, following a hospital stay related to a traumatic injury and an episode of delirium tremens. The sequence was selected by the researcher because it explored two subjects not yet addressed in the other video recorded clinical interviews: the exploration of past consumption habits and the exploration of change.

At the start of the sequence, the clinician (C2) explores the patient’s alcohol consumption prior to his hospital stay. As the patient recounts his previous habits, C2 punctuates his patient’s words by a simple “okay.” This specific part of the first sequence will support the discussion on disgust further in the process.

P: Well, I used to start drinking at 6p.m.

C2: Okay. And this is a change in your consumption, that is to say before the hospital stay... that is to say that before the hospital stay you used to drink also during the day?

P: Ah yeah yeah! Before the hospital stay, I used to start the day with a whiskey shot. Because that was [P shows his throat with his hand], how can I say, what used to clear my throat the best.

C2: Okay.

P: So I used to start with a shot of whiskey and then after I used to drink my coffee and then after go shopping. At around 10a.m. I used to open my bottle of Rosé [wine]. And I used to open my bottle of Red [wine] at around 7p.m., there I used to eat, go to bed and that was it. And then it started again the next day.

### Step 2C: Simple SCI

As exposed above, no special attention to this part of the clinical interview is yet paid in simple SCI. While viewing the first sequence of own clinical interview, C2 does not react while watching himself listen and consent to the patient’s past consumption habits. He goes on watching the sequence and stops the recording further to comment on his reaction when confronted with confused memories related to the patient’s delirium tremens.

### Step 2D: Cross SCI

During cross SCI, C2’s colleague (C1) watches and comments on the video montage of C2’s clinical interview in the presence of C2. At the first sequence, C1 stops the recording when C2 acknowledges the patient’s story with a simple “okay.” She enhances C2’s reaction, which she perceives as non-judgmental, and explains alternative reactions that C2 could have had while listening to the patient’s story. This passage is a turning point and the dialogue starts from there.

C1: But even this “okay”, I find it very good. Because when he says that... well, he says he starts the day with a... by drinking whiskey. And then this is something that, well, it might surprise, it might disgust, it might make you judge, it might lead to many reactions. Well, he drinks whiskey as soon as he opens his eyes and then he drinks his coffee. Well, at the same time you say that he was very sick and that he was in intensive care. So this shows… This “okay”, it’s just that you have no judgment. Okay, your life was like that, your habits, that world you lived in. It seems very soothing to me to hear you.

C2 listens attentively and nods, but doesn't speak. The dialogue could have ended here.

As if something was left aside in C1’s first comment and C2’s silence, the researcher relaunches the discussion by asking C1 about her own reaction during the viewing. Clinician 1 introduces the theme that will be at the heart of the following exchange by expressing her own reaction of disgust. Then she mentions again the specific moment where she interrupted the viewing and values once again her colleague’s attitude and compares it to her own reaction.

Researcher: You talk about the reaction that these words could provoke. What was your own reaction?

C1: By hearing, by observing?

Researcher: Yes.

C1: I was disgusted. I had the image, well the taste of whiskey, I mean how it can be to have the taste of whiskey for, as he says, “clear your throat”. I mean the words he uses can be very physical. Uh... at 7 a.m. Well it was really almost a physical disgust when I heard that. And then, hearing C2 just say “okay”, well I think it must be very calming for someone who is used to suffer.

Again, C2 listens and nods but does not react spontaneously. The researcher uses C1’s comment to get C2 to react on the way he handles this part of the clinical interview. Clinician 2 briefly evokes a similar reaction to C1, without explicitly naming disgust.

Researcher: What do you think about what C1 says? Does it mean anything to you?

C2: Yes. He is a patient…I understand that he can provoke this kind of reaction and I think that I… I had them [these reactions] myself. He’s a very lonely patient, who is... who doesn’t speak to anyone. At his place... I imagine it must be a little catastrophic in terms of hygiene. He is neglected, so this is it, I understand. That may provoke this [to be disgusted].

In order to carry on the discussion and focus on clinical practice, the researcher explored possible clinical responses to disgust. Clinician 2 and C1 present possible strategies.

Researcher: How do we cope with disgust? [Long break. C1 and C2 raise their eyebrows]

C2: We want to make it conscious, to know ... how to recognize it.

C1: [We want] to tolerate it, first of all.

C2: And this, in a context of… vulnerability, fragility. It is disgusting because it is the disease, it is the deformity, it is... the decline. And then to tolerate it, too. In my opinion, every time we see a patient, it’s a bit of a staging. We use our interpersonal skills and then we have to play a role of... to not play disgust. Yes, it is really as if we were actors but in the sense of... consenting to what the patient could... malleable tools, we are malleable for the patient.

Then C1 choses to progress in the viewing of the sequence and comments on the way C2 manages the patient’s confused discourse related to his episode of delirium tremens. But her final comment returns to the beginning of the sequence and her own reaction of disgust, as if she needed to explore it a little more.

C1: Until now, I mean... only these two and a half minutes, it impresses me [to see] how much violence there is, after all. These are violent scenes, to walk among dying people, to believe that… to believe that he was dead. At the beginning also… I mean, all this disgust at the beginning. It’s not easy, I imagine, to be in front of him.

Once again, the researcher uses C1’s assumption to bring C2 back to the clinical interview and the initial activity. C2 goes further in recognizing his own disgust and acknowledges the difficulty in taking care of his patient. At that moment, C1 and C2 share a common clinical reality.

Researcher: What do you think about what C2 says: "It's not easy to be in front of him"?

C2: I didn’t expect to be in front of him, in the sense that I had also said that I didn’t expect him to come for consultation. When he accepted the [medical] follow-up, I said to myself: Well, he’s not going to come. Was it also a desire that he didn’t come? [C2 smiles] And then I chose this patient a bit by default, he was the first who accepted [to be video recorded in the context of the research]. I wouldn’t have chosen him as a model patient or as a patient I wanted to show off. I also recognize this repulsive aspect that he can have in what he says, in how he presents himself. So, I agree [that] it’s not easy.

C1 goes on and generalizes C2’s comment to all psychiatric and substance use disorder patients. Then C1 tries to explain more about her own disgust and related clinical difficulty. At this point, discourse on disgust evolves and indirectly addresses its moral dimension.

C1: But I get the impression, when you said before, well... that this is the majority of our patients. Patients who don’t really appeal to us, who disgust us or say very violent things to us or very... Who disgust us in the sense that there is a lot of violence in what they experience. Also in all that is very physical in addiction… there are many things that are, in some ways, repulsive. Especially these patients that we meet… to get to intensive care it is necessary… that is to say they are also often in pronounced states of neglect.

One last time, the researcher brings C2’s attention back to the initial clinical interview and makes him watch himself. By valuing his profession and more indirectly his work, C2 joins C1’s initial valorization of his attitude, as if he realizes that he managed to overcome disgust. At this point, a transformation in the professional’s perception of his activity happens.

Researcher: And if I go back to what you said about this clinical interview and this patient: you said that you did not necessarily expect him to come, and that you maybe also did not want him to come. [C1 nods]. And then, in the end, when you see yourself working with him...

C2: Especially with the sentence that he says at the end, it touches me and it… Hmm, it makes me think it’s… noble [C2 smiles], there is a nobility in this work. In the sense that I feel like this patient has little opportunity in his life to chat with someone like that. And then being there for that… being used for this… it is already useful in my opinion. It’s very humane, just the human contact. Therefore, being able to overcome and to be aware of that disgust and be there nevertheless, it is rewarding. What we do is noble, that’s very romantic [C2 laughs].

After a brief silence, C1 restarts the video. Subsequent exchanges only concern other sequences.

### Step 3A: Group Interview

An initial analysis of the material collected highlighted disgust as one of eight emerging themes. This result was presented to the pair of professionals within a group interview in the form of a video montage which depicted the evolution of discourse as we have just presented it in this section. The aim of the session was to co-approve this theme as a significant research result and to further elaborate related clinical challenges and responses with the professionals.

After watching the montage, the pair validated the theme of disgust as a central difficulty in their clinical practice.

C1: I think it’s [disgust] something that deserves to be in the central themes. Precisely because it is recurring, it is not just that patient.

A little further in the discussion, C1 evokes the trigger action in the clinical interview (i.e., “okay”). In this passage, we move away from disgust itself to identify more precisely the implied clinical challenges and required competences to deal with disgust: accepting disgust as part of clinical practice.

C1: And it’s also, at the very beginning of the video sequence, it’s also there... well we say it later too, how [can we] accept it all, how [can we] handle it all? Well, the fact that C2 says “okay”, that’s what we finally accept, working in these... that’s the rules of the game after all, that it's gonna be disgusting, that it's gonna be violent. The question is how do we... live through as a therapist in front of... so that we can help them afterwards? [We must] already be able to say it, this “okay”, without it being a lie. To really be able to say it honestly, that we are okay with that, it is already very demanding. And this is the prerequisite to be able to work afterwards, I think.

### Step 3C: Feedback Questionnaire

Clinician 2 directly alludes to the theme of disgust and the related discussion in the feedback questionnaire. In his comment, he demonstrates the impact of the general process in terms of reflexivity and highlights the discussion on disgust as one relevant moment. The notion of “transparency” suggests that the methodological setting has allowed the emergence of a usually unspoken theme.

C2: The feedback is positive. The experience was very rich. Getting a reflection of one’s own clinical activity allows to examine daily practice subtleties that otherwise escape. These subtleties are of major importance because they are at the center of the relational issues that shape our practice. A particular moment: the discussion with [C1] when watching my patient’s video, where we discussed disgust with great transparency and clinical interest. It is a unique moment in everyday practice.

### Step 3D: Research Group meeting

Reporting this specific result to the research group had two outcomes. First, the discussion made it possible to better describe the emergence of this theme and to highlight the underlying methodological process. Second, the discussion initiated secondary analyses. The interpretation of disgust as a moral challenge initiated the development of a final system for categorizing emerging themes, which included moral challenges, epistemic challenges, technical challenges, and institutional challenges as main categories.

## Discussion

### Challenges

As members of the hospital and the healthcare research community, we took advantage of facilitated access to clinical settings and a large institutional network to implement this design. This connection facilitated specific methodological steps, such as recruitment of participants and anticipation of ethical issues specific to the use of highly confidential medical data. Further use and communication of results through the institution was also made easier. Despite these observations, applying CSC in a clinical surrounding was challenging.

First, researchers have to deal with a particularly labor-intensive, time-consuming, and complex methodological process. Coordinating the overall research, editing multiple video montages between steps, combining use of audiovisual and written material, and scheduling interviews and group meetings demands an inordinate amount of time and resources. Although we are used to working with qualitative material, we recognize that the volume of collected data for this project is impressive and out of step with the number of included participants. However, this challenge was partly related to a first use of CSC. Future application of the method will definitely be simplified.

Dealing with highly confidential material and medical data was a second challenge. Even though the patients were not the research participants, their formal consent was required, and patients’ faces and voices have to be blurred for public presentations as for any research using medical data. Mostly, ethical considerations led us to select outpatient activities with a more confidential setting for video recording than inpatient activities (i.e., one patient per consultation room, closed consultation rooms, and no interference with daily hospital activity). Ethical considerations might restrict access to those portions of clinical activity involving patients and must be considered in future research projects in clinical surroundings.

Another challenge concerned the use of CSC for those unfamiliar with the process. In contrast to other interview types based on dialogue between a researcher and a participant, such as confrontational interviews and deliberative interviews (Berner-Rodoreda et al., 2020), CSC is based on a participant’s dialogues with oneself and peers. Researchers must be able to partially withdraw. They must limit their interventions to creating dialogue instead of asking questions to understand the content of the dialogue. During simple SCI and cross SCI, participants choose when to stop a video, which footage to focus on, and what part of the activities they wish to discuss. And during results restitution, they are free to reshape and label results in their own way. Researchers have to put aside part of their own questions, observations, and interpretations. The leading researcher experienced feeling a loss of control during the first two simple SCIs; she was reviewing video recorded activities without being free to point to her own questions. She simply had to trust the process, and stick to a path that was foreign to her. Her experience demonstrates the need to be particularly well-prepared and confident about how CSC interviews can be productive. As for the research results, they support that this challenge has been successfully completed.

One final challenge and perhaps the strangest part of adapting CSC to a clinical setting was to analyze an intersubjective activity between a professional and a patient by focusing on the professional’s activity. In the approach proposed by the method, the clinician actively participates while the patient’s complementary input and experience are ignored. The patient is not included in the process, even though the patient is the very condition of the activity. Applying CSC to a clinical encounter made it hard not to be tempted to choose an alternative design that allows cross SCI between a clinician and a patient based on their common activity, namely the clinical interview. But this development, which echoes Wyer’s interesting use of video-reflexive ethnography (Wyer et al., 2017), would introduce a significant departure from the original method. The professional-oriented approach of CSC is actually a major contribution, since it makes possible an exploration on clinical practice in terms of difficulties, uncertainties, and moral challenges *among peers*.

### Limitations

Major limitations pertain to our adaptations from the original method as developed by the Activity Clinic framework. A main one is the composition of our research group.

The group met the prerequisite of “building a collective of professionals around the concerns of the profession” (Kostulski, 2010, p. 31), but it was not a collective of peers since hierarchical level and interdisciplinary standards were not representative of our clinician pairs. Our research included pairs of nurses and medical doctors. However, nurses were not included in the research group, which naturally limited interdisciplinary input and data discussion. We partially overcame this limitation by including nurses’ input in the complementary data collection of the general study. However, future research should definitely pay attention to establishing an inclusive research group that is able to represent and express the interests of each.

Due to the complexity of organization in a clinical setting (e.g., irregular work schedules, emergencies, and frequent turnovers) and to our interdisciplinary and multi-site perspective, we did not bring together the three pairs and the researchers in a single analysis group. Preliminary discussions and results restitutions were conducted separately within the research group and within each pair. The lead researcher was the liaison between the two groups. This particular departure from the original method relieved the system and facilitated the organization of meetings. But it represents the most evident adaptation compared to the original method. In our design the third phase of dialogue, that is, the group interview, is an extension of cross SCI rather than a new dialogical setting with a larger group of peers. This adaptation restricted the multiplicity of dialogical settings required by CSC and contributed to another limitation, that is, participants’ partial elevation to co-researcher status. As other contemporary methodological approaches that develop within a new politics of research participation and knowledge production (Cornwall & Jewkes, 1995; Smit et al., 2021), CSC claims a process of co-researching and associates researchers and professionals in a co-construction and co-analysis process (Clot et al., 2000; Kloetzer, 2018). Although the expected participation of clinicians was achieved during the first two phases, clinicians tended to be less involved when finalizing and approving results during group interviews. Sharing analyses with other pairs of professionals and returning results personally to the investigators may have improved distribution of the results and should be part of further use of CSC. As recommended by the original theoretical framework, CSC should also concentrate on areas where specific questioning is grounded and carried from the start by field professionals (Kostulski, 2010).

Overall, despite encountered challenges and limitations, applying CSC in a clinical context was very positive. Some strengths of the method and fields of application are presented below.

### Research Perspectives: The Emergence of Tacit Knowledge and Alternative Paths of Action

Through a specific reflective activity based on audiovisual and grounded in the field material, CSC allowed for an exploration of clinical difficulties experienced by nurses and medical doctors. The emergence of one theme, disgust, was given as an illustration of the methodological process. This specific clinical difficulty and its close connection with stigmatization and negative attitudes are demonstrated within specific nursing and medical contexts (Finnell, 2018; Kaiser et al., 2019; Reynolds 2013; Schnall et al., 2008). The intervention of moral judgments in the clinical relationship (Hill, 2010), and more specifically in the fields of psychiatry and addiction medicine (Howard & Chung, 2000; Shaw, 2004), is also documented. What we retain from this specific illustration is firstly that the opportunity to recognize disgust was created by the method, whereas it could have gone unnoticed. Secondly, we retain that an opportunity is created by CSC to reflect on own clinical difficulties. Results emerge through a technique reminiscent of Schön’s reflective model (Schön, 1983) by acknowledging the interaction between tacit knowledge and actions, and by bringing to the surface new understandings that shape our actions. The methodological process not only allowed to identify a moral sentiment (Smith, 1759/2002) but it also made it possible to understand how a moral sentiment induces a judgment and becomes a difficulty for clinical practice. Clinicians’ awareness of disgust made it possible to reflect on related clinical challenges (e.g., caring for a patient who disgusts us/that we judge); on own resources (e.g., recognition of own disgust/moral judgement and acceptation of disgust as part of clinical practice); and on alternative paths of action (e.g., “to say it, this *okay*, without it being a lie”) that clinicians can “embody in further actions” (Schön, 1983, p. 50). Thus, one strength of the method was not only to allow the emergence of tacit clinical difficulties but also to initiate a transformation of practice.

As expected, CSC probably provided access to information that would have escaped clinical interaction observations or the discourse on clinical practice, had it been a semi-structured interview. Most commonly used qualitative methods have great potentials in generating knowledge and stimulating uses of these have demonstrated, for example, the interest of participant observation for exploring clinical practice (Savage, 2000), the transformative potential of focus groups (Hyde et al., 2005) or the development of participant reflexivity through interview (Perera, 2020). However, our results support that CSC heightened the reflective potential of the research process. Discussing insecurities, recognizing areas of incompetence and disclosing errors is particularly confronting for actors of modern medicine (LaDonna et al., 2018; Rosenthal, 1995; Wu, 2000), whose core symbol is competence (DelVecchio Good, 1995). Voicing own counter-attitudes and moral challenges appears to be even more difficult (Kaiser et al., 2019; Martin et al., 2002) and requires adapted research or educational settings (Ballon & Skinner, 2008; Skinner et al., 2009). Our findings suggest that CSC is a one of them and that it is a good method to favor access to “this ‘hard to say’ with which we could maybe do something different than what we do” (Clot, 2005, p. 43).

The use of CSC appears to be particularly relevant for the field of addiction-related practice to explore lower regard for working with patients with substance use disorder and help nurses and other healthcare providers question their own relation to addiction. CSC may be of particular interest in clinical setting with patients perceived as “difficult patients” (Koekkoek et al., 2011), “dirty work patients” (Shaw, 2004) or “problem patients” (May & Kelly, 1982), to work on the professionals’ negative attitudes, stigmatizing reactions or any other avoidance mechanisms that directly affect quality of care and more indirectly, health equity. More generally, CSC has the potential to elicit the unspoken part of clinical activity and is of specific interest for any research project that aims to explore values, social representations, hidden assumptions, unconscious bias, moral feelings, or other implicit knowledge that underlies the experience of care.

Based on these considerations, CSC might be of specific interest for nurses. The ability to provide patient-centered care and to establish a trusting therapeutic relationship are key components of nursing expertise. Understanding and working on the numerous challenges that put these abilities at risk is central to improve care for patients as well as the experience of care for nurses. The reflective activity proposed by CSC and its capacity to address the mechanisms that encourages avoidance, such as moral issues or stigmatizing reactions to physical, behavioral or cultural difference, can definitely help develop and maintain a therapeutic alliance. Nursing contexts where the therapeutic relationship with patient and family is particularly challenged, including mental health care (Valente, 2017), critical care (O’Connell, 2008) or palliative care (Wallace, 2001) could benefit from the method.

The methodology of CSC is an interesting tool for any institutions, clinical teams or professionals that wish to explore and develop their clinical practice, and improve care for patients accordingly. The applicability of this method in a diversity of international settings may raise some issues, though. Projected obstacles include poor access to video device and editing software or related additional equipment costs. In addition, a special attention has to be paid to cultural and institutional habits regarding use of audiovisual data and more generally to cultural or individual relationship to images.

### Educational Perspectives: A Comparison With Clinical Supervision

The results indirectly highlighted CSC’s potential as an interesting practice-based learning setting and initiated a comparison with clinical supervision settings. By proposing a group reflective activity based on clinical material, CSC echoes various modalities of clinical supervision, such as peer supervision (Owen & Shohet, 2013), Balint groups (Balint, 1957; Salinsky et al., 2006) or any other collaborative learning groups whose purpose is to discuss clinical challenges based on the assumption that clinical practice uncertainty deserves “considered reflection” (Launer, 2015, p. 473). The resemblance is based on a common understanding of the activity as “one of the possible activities in all of those that could have been or could be accomplished” (Clot, 2006, p. 170), as well as a common intention to question daily activity and highlight alternative ways of practicing. Our experience of CSC confirmed these theoretical premises and helped clarify three distinctive features.

The first relates to *temporality*. Cross self-confrontation is based on a slow immersion consisting of distinct phases, distinct settings, change of protagonists, multiple viewings, and transition from video to written material. This differs from the more reactive temporality of clinical supervision. But mostly, while clinical supervision’s reflective process evolves over time through a variety of new material that is reported when new clinical experiences arise, CSC remains focused on a single material over the whole reflective process. This difference is partly linked to the primary focus of each approach. In CSC, material reported in the form of clinical activity is first and foremost a methodological means taking the form of reflective support in a context of research with extendable temporality, whereas clinical supervision depends on a time-bound framework that meets a training objective.

The second distinctive feature relates to *multiplicity*. Changes of protagonists and multiplicity of dialogues are central in CSC. Use of dialogue between professionals to stimulate participant’s inner dialogue (Kostulski & Kloetzer, 2014) echoes individual and group clinical supervision. Similarly, the analysis of a person’s way of acting through the eyes of a peer that is proposed by cross SCI echoes group clinical supervision. However, there are some major differences; clinical supervision does not rely on a successive application of individual and group settings, whereas CSC takes advantage of crossing viewpoints. By diversifying settings and the recipients of dialogue, scrutiny on practice is renewed at each step. In our research, switching from the individual to the collective was the facilitator of the emergence of disgust and brought to the forefront what the clinician had initially kept silent.

The third distinctive feature relates to *expertise*. Cross self-confrontation interviews aim at reinforcing peer learning (Kloetzer et al., 2018) and differs from clinical supervision because of the absence of an expert or a facilitator relying on own professional experience in promoting reflective practice. In our research, the researcher was not invested as a representative of the clinical community and the profession’s good practices; this probably favored a non-judgmental environment promoting dialogue regarding experience of care and related difficulties.

Observations of CSC as an educational tool in clinical settings suggest that CSC is an interesting method to question practice and to improve self-reflection, which differs from clinical supervision in its various forms. Although it is difficult to document, the assumption is that CSC’s temporality but mostly its multiplicity of dialogical settings will result in deeper self-reflection. And we believe that the absence of clinical expertise in discussions of clinical practice may lead to discussing issues that are not normally shared with clinical facilitators. However, the long term effectiveness of CSC compared to traditional clinical supervision settings has to be tested and the levels of reflection induced by the two processes have to be compared. Specific target audiences, particular fields of clinical practice and potential required adaptations so that it can be used easily in a learning context must also be defined, including formal evaluation of CSC as a teaching vehicle for nursing practice specifically.

## Concluding discussion

The intention of this article was to present and discuss a novel application of CSC in a clinical setting. Through a specific process of co-analyzing video recorded clinical interviews, CSC made possible an exploration of addiction-related clinical difficulties experienced by hospital-based staff and stimulated self-reflection among nurses and medical doctors. We posit two major strengths: the method’s capacity to question professionals both individually and collectively; and the capacity to elicit tacit knowledge of daily clinical practice. We believe the use of CSC is an opportunity for developing grounded, collaborative and performative research projects in clinical and hospital settings. We encourage further use of CSC in nursing and medical contexts, as well as the formal evaluation of CSC in a nursing educational context.
